# Investigation of the efficacy of low-frequency repetitive transcranial magnetic stimulation on upper-limb motor recovery in subacute ischemic stroke without cortical involvement: a protocol paper for a multi-center, double-blind randomized controlled trial

**DOI:** 10.3389/fneur.2023.1216510

**Published:** 2023-08-25

**Authors:** Hee-Mun Cho, Seungwoo Cha, Min Kyun Sohn, Sungju Jee, Won Kee Chang, Won-Seok Kim, Nam-Jong Paik

**Affiliations:** ^1^Department of Rehabilitation Medicine, Seoul National University College of Medicine, Seoul National University Bundang Hospital, Seongnam-si, Republic of Korea; ^2^Department of Rehabilitation Medicine, Chungnam National University College of Medicine, Chungnam National University Hospital, Daejeon, Republic of Korea

**Keywords:** stroke, transcranial magnetic stimulation, rTMS, upper limb motor function, recovery of function, rehabilitation, randomized controlled trial

## Abstract

**Introduction:**

The incidence of stroke is increasing steadily due to factors such as population aging. Approximately 80% of stroke survivors have motor disorders affecting their daily lives. Repetitive transcranial magnetic stimulation (rTMS) has been reported to maximize functional recovery after stroke along with exercise intervention in upper limb rehabilitation treatment. However, whether rTMS affects the recovery of upper limb function in patients with stroke remains unclear. Therefore, in this trial, we will investigate the efficacy of low-frequency rTMS in patients with subcortical and brainstem ischemic stroke.

**Methods:**

This study has been designed as a multi-center, double-blind, randomized controlled trial to compare the efficacy of low-frequency rTMS over the contralesional M1 with sham stimulation. Overall, 88 participants will be allocated to the intervention or control group in a 1:1 ratio, with stratification according to their initial upper extremity Fugl-Meyer assessment (UE-FMA) score. The participants will receive either 30 min of real rTMS (intervention group) or sham rTMS (control group), followed by 30 min of occupational therapy for 10 consecutive workdays. All the participants will receive the same amount of rehabilitation therapy throughout the intervention period. Evaluations will be performed at baseline (T0), at the end of treatment (T1), and 4 weeks after the end of treatment (T2), including the box and block test (BBT), UE-FMA, Korean version of the Modified Barthel Index, and NIH Stroke Scale scores, Finger tapping test, Brunnstrom stage, modified Ashworth scale, and grip strength. The primary outcome will be the change in the BBT score between T0 and T2.

**Conclusion:**

This study will provide evidence on the efficacy of low-frequency rTMS in motor function recovery of the upper limb in patients with subacute, subcortical, and brainstem ischemic stroke.

**Clinical trial registration:**

ClinicalTrials.gov, identifier [NCT05535504].

## 1. Introduction

Stroke occurs in more than 12 million people worldwide every year, and its incidence is steadily increasing owing to various factors, including population aging (1). Approximately 80% of stroke survivors have motor disorders that affect their daily lives (2). Furthermore, 60–70% of the patients with stroke experience upper extremity motor function damage during hospitalization, and approximately half (33% of all strokes) of them are classified as having severe paralysis. Additionally, 50–70% of stroke survivors suffer paralysis of upper extremity motor function 2–4 years after stroke (3).

Various treatments have been attempted to restore upper limb motor function after stroke, with upper limb rehabilitation treatment, including various physical training approaches (work therapy, muscle strengthening, etc.), currently being applied as a standard treatment for upper limb motor function recovery after stroke (4). These upper-limb rehabilitation treatments induce continuous cortical reconstruction in both hemispheres and promote adaptation to cortical maps, resulting in neuroplasticity after stroke, which benefits functional recovery with longer repetition or intervention periods. However, the extent of recovery of damaged brain tissue and its function using this method is limited. Therefore, many patients have limitations in function due to the aftereffects of stroke, which leads to an increase in social costs (4, 5).

Recently, several non-invasive neuroregulatory intervention studies have reported maximizing functional recovery after stroke in upper limb rehabilitation (5); several studies have reported that repetitive transcranial magnetic stimulation (rTMS) with rehabilitation training such as occupational therapy increases functional recovery (4). Chang et al. (6) investigated the effects of high-frequency rTMS (10 Hz) on the affected hemisphere’s primary motor cortex by comparing it with sham rTMS in patients with subacute stroke who underwent motor training. The results revealed that the group that received the real rTMS showed greater recovery in the sequential finger motor task, indicating the positive impact of rTMS on motor function improvement (6). Di Lazzaro et al. (7) conducted a study comparing the effects of inhibitory theta burst stimulation (iTBS) and sham TBS administered prior to physical therapy in patients with chronic stroke. The results revealed that the iTBS group showed significantly higher scores on the Jebsen-Taylor Test at 3 months post treatment, compared with the sham group (7). rTMS is a treatment method that repetitively stimulates the cerebral nerve area by inducing current to the cerebral cortex, by forming a magnetic field similar to that of Magnetic Resonance Imaging (MRI) in the cerebral cortex. It is a non-invasive and safe method that is widely used not only for patients with stroke but also for neurostimulation in patients with depression, Parkinson’s disease, Huntington’s disease, and cerebral palsy (5).

Representative rTMS protocols include high-frequency rTMS, which is mainly implemented in the ipsilesional primary motor cortex, and low-frequency rTMS, which is mainly implemented in the contralesional M1 (8). Low-frequency rTMS over the contralesional primary motor cortex (M1) has been used to suppress excessive inhibition of ipsilesional M1. That is, it is applied as an attempt to reduce interhemispheric inhibition (IHI) (9). Low frequency rTMS has less impact on motor behavior and reduces risk of seizures in healthy cortical spine systems (8, 10). Therefore, most studies on motor recovery after stroke have applied low-frequency rTMS over the contralesional M1 region.

Research on the effectiveness of rTMS remains controversial. Hsu et al. (11) performed a meta-analysis of 34 existing studies in 2012 and confirmed the significant effects of rTMS on upper limb motor function recovery in patients with stroke. In contrast, Graef et al. (4) investigated the effects of rTMS combined with upper limb training vs. sham rTMS combined with upper limb training on upper limb recovery after stroke in 2016 and concluded that there were no significant differences between groups. Harvey et al. (12) reported a clinical study comparing the effects of rTMS and sham rTMS on the functional recovery of the upper extremities of stroke patients in 2018, and found no difference between the rTMS and sham rTMS groups. Therefore, it is unclear whether rTMS affects the recovery of upper limb function in patients with stroke. Harvey et al. (12) suggested that no significant effect was confirmed because the effect of rTMS may vary depending on the location or severity of the stroke. Graef et al. (4) suggested that the heterogeneity of occupational therapy between studies was the reason why a significant effect of rTMS was not confirmed in the meta-analysis.

Kim et al. (13) conducted a randomized controlled trial to determine the efficacy of low-frequency rTMS on the contralateral M1 combined with occupational therapy in patients with stroke. Although the study did not show a significant difference between the rTMS and sham groups, subgroup analysis based on cortical involvement revealed that low frequency rTMS on contralesional M1 led to improvements in the Brunnstrom stage of hand function in the group without cortical involvement. This result led us to hypothesize that the type and location of stroke may affect the efficacy of rTMS warranting further investigation.

Therefore, a multicenter randomized controlled trial has been designed to confirm the effectiveness of low-frequency rTMS over the contralesional M1 in patients with subcortical and brainstem ischemic stroke.

## 2. Methods

### 2.1. Study design

In this prospective, multi-center, double-blind, randomized sham-controlled, superiority trial, the effectiveness of low-frequency rTMS over the contralesional M1 will be compared to that of sham rTMS in patients who require upper limb rehabilitation due to subcortical and brainstem ischemic stroke.

**Figure 1:**
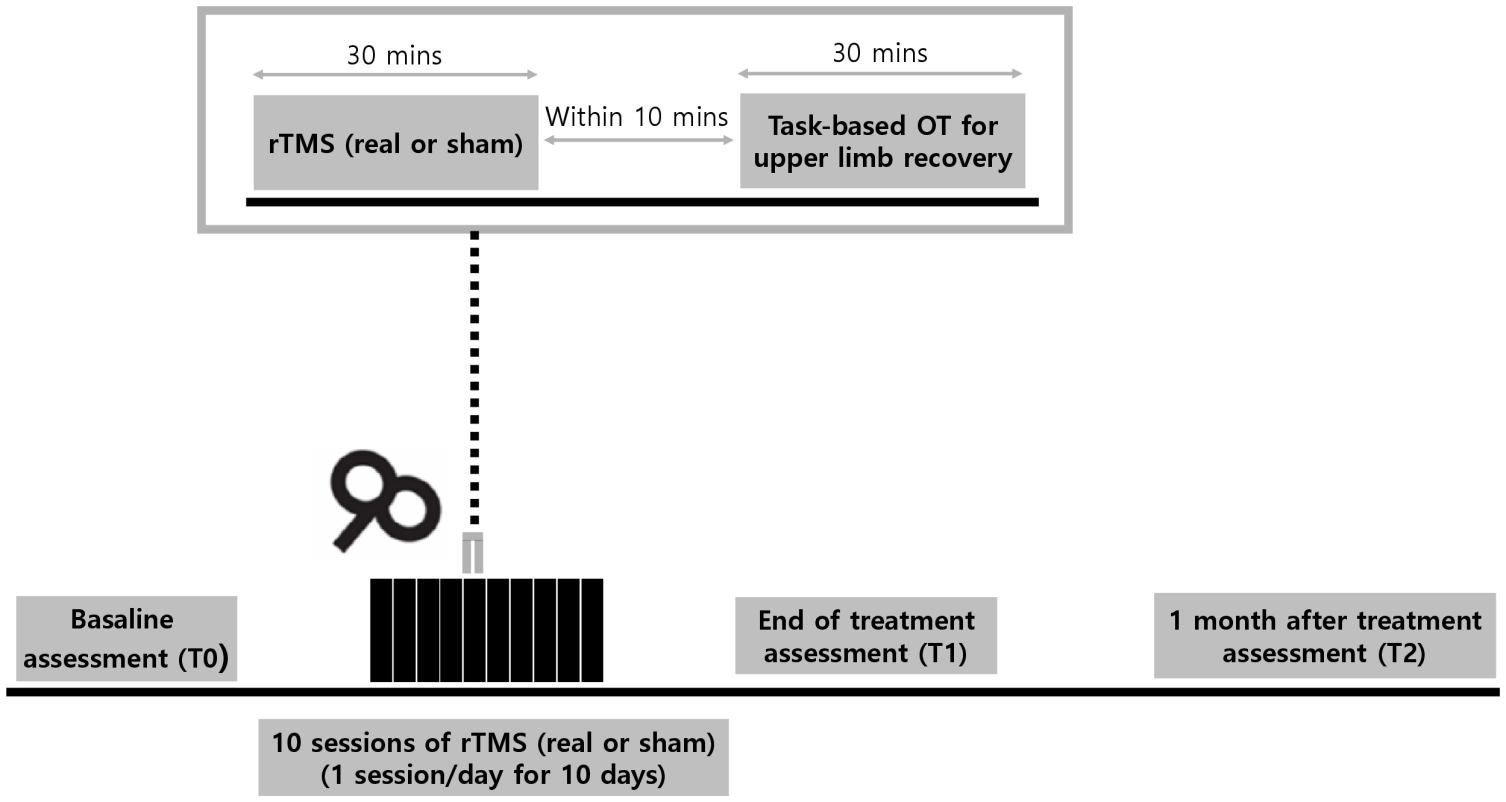
Experimental design. Participants will receive 30-min of daily real or sham repetitive transcranial magnetic stimulations (rTMS) over a period of 10 consecutive workdays. Task-based occupational therapy (OT) of 30 min for upper limb recovery will be applied within 10 min after rTMS session. Assessments will be performed at baseline (T0), end of treatment (T1), and 1 month (T2) after the last session of rTMS.

Patients admitted with acute stroke will be recruited from the rehabilitation ward. Eligibility will be screened by the research team and the patients will be randomly allocated to the intervention group (real rTMS) or control group (sham rTMS) in a 1:1 ratio, with stratification into two subgroups according to the baseline Upper Extremities Fugl-Meyer Assessment (UE-FMA) score. The baseline evaluation (T0) will be performed by a blinded evaluator after group allocation. All participants will receive 30 min of intervention (real rTMS in the intervention group and sham rTMS in the control group), accompanied by 30 min of occupational therapy (OT) after the intervention. The intervention will be provided to participants for 10 consecutive working days. All participants will receive the same amount of occupational and physical therapy (PT) consisting of two sessions of 30-min OT and PT daily during this period. The evaluation will be performed at the end of the intervention period (T1) and 4 weeks after the end of the intervention (T2). The doses of OT and PT will be monitored and documented by the patient/caregiver at T1 and T2. An overview of the study design and process is shown in Figure 1.

### 2.2. Participants and recruitment

In this study, the authors will enroll 88 adult participants, from two institutions recruiting 44 participants each: Seoul National University Bundang Hospital (Gyeonggi-do, Republic of Korea) and Chungnam National University Hospital (Daejeon, Republic of Korea).

The inclusion criteria are as follows: (a) between the ages of 19 and under 80, (b) within 90 days from first subcortical or brainstem ischemic stroke confirmed by Computed Tomography (CT) or Magnetic Resonance Image (MRI) (13), (c) UE-FMA score exceeding 15 points (14), and (d) Korean version of Mini Mental Status Exam (K-MMSE) score of 15 or higher. The exclusion criteria are as follows: (a) diagnosis of a cerebellar stroke, (b) history of cerebrovascular surgery, (c) wrist score of less than 1 and hand score of less than 1 (13), (d) history of psychiatric or neurological disease before stroke onset, (e) inability to undergo rehabilitation treatment for other reasons, such as medical problems, and (f) history of epilepsy or seizure disorder. The detailed inclusion and exclusion criteria are listed in Table 1.

**Table 1:** Major inclusion and exclusion criteria.

**Inclusion criteria**
Aged above 19 and under 80 Diagnosed of first-onset subcortical or brainstem ischemic stroke with imaging within 90 days Upper Extremity FMA score exceeds 15 points K-MMSE score of 15 or higher
**Exclusion criteria**
Among Upper Extremity FMA scores, those with a wrist score of less than 1 and a hand score of less than 1 Diagnosed with a cerebellar stroke With a history of cerebrovascular surgery With a history of psychiatric or neurological diseases before the onset of stroke Unable to undergo rehabilitation treatment due to other reasons, such as medical problems With a history of epilepsy or seizure disorder

FMA, Fugl-Meyer Assessment; K-MMSE; K-Mini Mental Status Exam.

All participants received detailed information about the trial and written informed consent was obtained. The research protocol has been approved by the institutional review boards of Seoul National University Bundang Hospital (IRB no: E-2010-642-001) and Chungnam National University Hospital (IRB no:2022-04-014). This study will be conducted in accordance with the Good Clinical Practice regulatory standards and the Declaration of Helsinki. The study protocol has been registered on the ClinicalTrials.gov, on September 10, 2022 (Identifier: NCT05535504).

### 2.3. Randomization and blinding

A list of random assignments will be prepared by a statistician who is independent of the research team. Within each institution, the participants will be stratified into two subgroups based on a baseline UE-FMA score of 28 using a stratified block randomization method with blocks of size 2 within each stratum, and the assignment ratio of the intervention and control groups will be maintained at 1:1. A random assignment list will be generated using SPSS version 23.0(IBM Corp., Armonk, NY, United States). The block size and seed number used will be randomly selected by a statistician responsible for the random assignment. Random assignments will be stored in opaque envelopes for each participant and delivered to the coordinator at the start of the intervention.

This clinical trial will be performed in a double (participant and evaluator) blind-fold manner, and the therapists who perform the treatment will also be blinded throughout the study. Owing to the nature of this clinical trial, the investigator who will perform rTMS will not be blinded to the assigned group.

### 2.4. Intervention

#### 2.4.1. rTMS intervention

**Figure 2:**
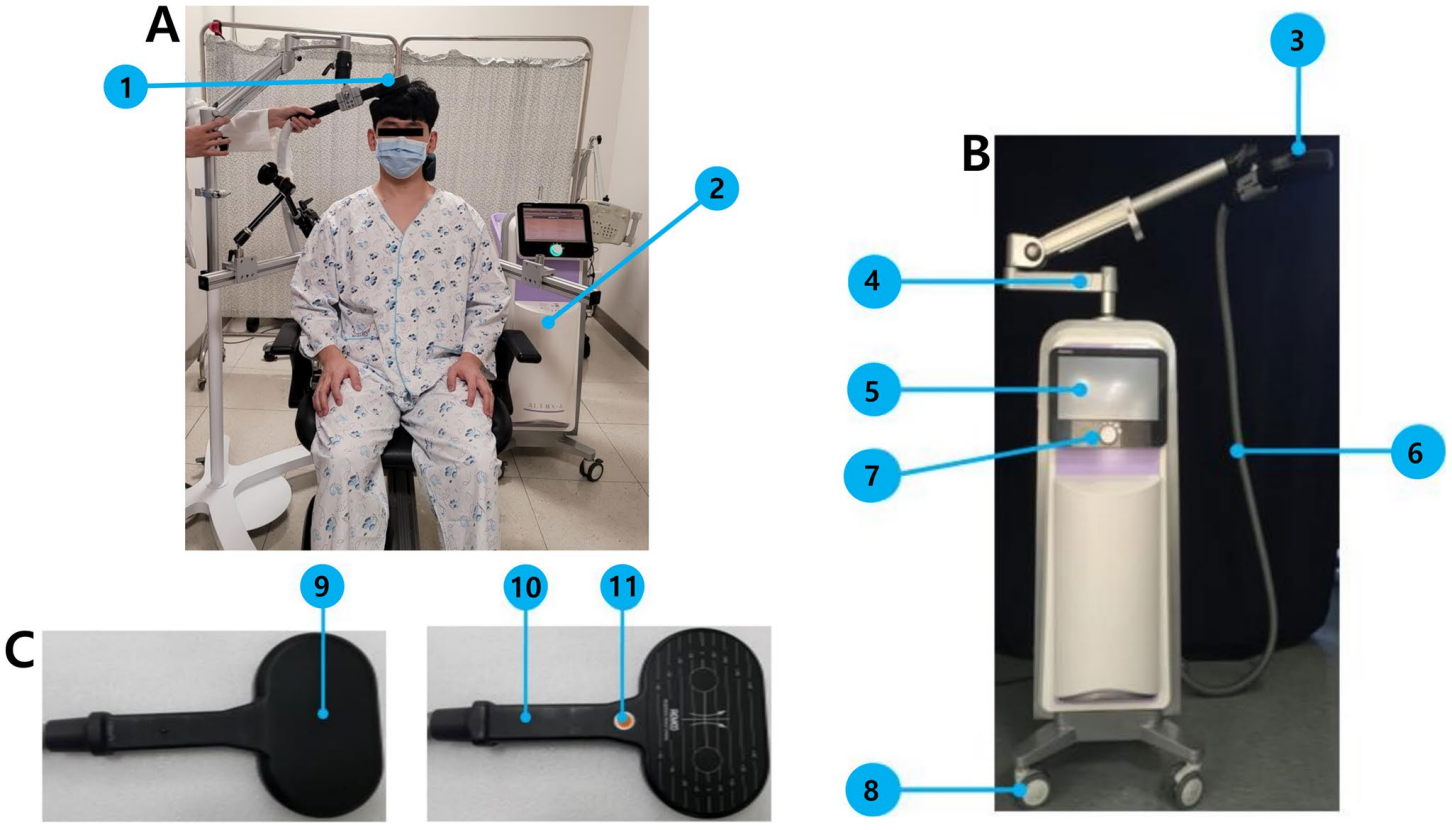
Instruments and implementation of rTMS in this trial. **(A)** Depictive figure of actual rTMS implementation, 1: Transducer (a 88-mm-diameter figure-of-eight coil), 2: main body of ALTMS-A. **(B)** ALTMS-A system, 3: Transducer, 4: transducer holding arm, 5: LCD touch panel, 6: transducer output cable, 7: controller, 8: carrying wheel. **(C)** Left: Transducer front, Right: transducer rear, 9: magnetic field generator, 10: handle, 11: stimulation switch.

Patients will be seated comfortably in a chair during rTMS. The resting motor threshold (RMT) for the first dorsal interosseous muscle of the unaffected side will be measured over the contralesional M1 (c3 or c4 according to the 10–20 system). The RMT is defined as the minimum stimulation intensity required to evoke a response of at least 50 μV in at least 5 out of 10 consecutive stimulations (15). In each session, in the intervention group, the contralesional M1 will be stimulated through a 88-mm-diameter figure-of-eight coil powered by an ALTMS-A (Remed, Seongnam-si, Republic of Korea) with a frequency of 1 Hz and an intensity of 100% of the patient’s RMT measured in the contralesional M1 for 30 min, to achieve 1,800 stimuli per session. The stimulation intensity and duration were determined based on previous studies on low-frequency rTMS (16) and safety guidelines (8). For the control group, sham rTMS will be applied, and the coil will be positioned perpendicular to the scalp on the contralesional M1 with the same intensity and frequency as that in the real rTMS. The details of the rTMS system used in this trial are shown in Figure 2.

##### 2.4.1.1. Implementation of occupational therapy

OT will be provided twice a day for 30 min each, starting within 10 min after rTMS in both the intervention and control groups between T0 and T1. Participants will receive one-on-one OT with an occupational therapist, comprising task-oriented therapy, including shaping exercises. The difficulty will be tailored to each participant and will be gradually increased according to the improvement in the motor function of the paralyzed hand. Therapists will be blinded to the participant groups throughout the trial. Participants will be provided with a rehabilitation diary, and the therapy dosage will be recorded during the period between T1 and T2. In the rehabilitation diary, the date and duration of rehabilitation treatment in minutes will be recorded as items of occupational therapy, physical therapy, or other treatment.

### 2.5. Outcomes

#### 2.5.1. Primary outcome measure

Primary outcome is the amount of change in the number of Box and Block Test (BBT) (17) at T2 compared to that at T0 (3, 5, 13, 18). The BBT is a manual dexterity test that has been used to evaluate physically disabled individuals. The BBT consists of a partition and wooden box divided into two compartments containing 150 blocks. Participants will be asked to move as much as possible, one at a time, from one compartment of the box to another of the same size, within 60 s. The BBT score is the number of blocks the participant has moved (17).

#### 2.5.2. Secondary outcome measures

Several secondary outcome measures will be assessed (Table 2), including: changes in the number of BBT at T1 compared to those at T0; UE-FMA (19) score changes at T1 and T2 compared to those at T0 (3, 13, 20); changes in the Korean version of Modified Bartherl Index (K-MBI) (21) score at T1and T2 compared to those at T0 (13, 22); changes in the NIH Stroke Scale (NIHSS) scores (23) at T1 and T2 compared to those at T0 (12, 13, 24); the finger tapping test (FTT) (25) (participants tap their index fingers as quickly as possible within a 10-s time interval, and the number of times they tap their fingers becomes an FFT score) score changes at T1and T2 compared to those at T0 (13, 24); the Brunnstrom Stage (26) changes at T1and T2 compared to those at T0 (13); the Modified Ashworth Scale (MAS) score changes at T1and T2 compared to those at T0 (13, 22, 27); and the degree of change in grip strength (hand grip, pinch grip, lateral pr, and three-jaw chuck) measured using the JAMAR dynamometer (Performance Health Supply Inc., Cedarburg, United States) at T1 and T2 compared to that at T0 (3, 13, 22).

**Table 2:** Timetable and measures to be made.

	Intervention period		4 weeks after EOT (T2)
	Baseline (T0)	EOT (T1)	
**Primary outcome measure**			
Box Block Test	√	√	√
**Secondary outcome measures**			
Upper extremity FMA	√	√	√
K-MBI	√	√	√
NIHSS	√	√	√
Finger Tapping Test	√	√	√
Brunnstrom stage	√	√	√
Modified Ashworth scale	√	√	√
Grip strength	√	√	√

EOT, end-of-treatment; FMA, Fugl-Meyer Assessment; K-MBI, Korean version of the Modified Barthel Index; NIHSS, National Institutes of Health Stroke Scale.

### 2.6. Sample size

According to a previous study (13), the average change in BBT at 1 month after baseline was confirmed to be 17.40 (9.80) in subcortical and brainstem stroke subjects who underwent rTMS and 10.90 (10.30) in subjects who received sham stimulation. The average change in BBT in the intervention and control groups in this clinical trial is also expected to be similar, and the standard deviation is assumed to be a large value of 10.30 for the conservatively setting. Assuming a significance level of 5% and a power of 80% under a BBT average change of 6.50 (intervention group: 17.40, control group: 10.90) and a standard deviation of 10.30, the number of subjects calculated is at least 40 per group; therefore, a total of 88 subjects will be recruited, considering a 10% dropout rate.

### 2.7. Data management and sharing

All participants’ data will be collected by research team members and will be stored as form of an Electronic Case Report Form (eCRF) in secured network cubeCDMS^®^ system (CRScube Inc., Seoul, Korea). The backup database will be updated regularly, and only the research team will have access to it. Case report forms will be stored in the research team’s locked cabinet. After completing the eCRF entry, the data will be frozen, and locked if there are no abnormalities. The collected data will be recorded on a CD and sent to Seoul National University Bundang Hospital and Chungnam National University Hospital.

### 2.8. Primary hypothesis and data analysis

#### 2.8.1. Primary hypothesis

The primary hypothesis of the study is that the change in the number of BBT, at T2 compared to that at T0 in the intervention group that received real rTMS and occupational therapy, would be significantly greater than that in the control group that received sham rTMS and occupational therapy.

#### 2.8.2. Analysis of demographic and other underlying data

Demographic and other characteristics will be analyzed using a group targeting the full analysis (FA) set. Analysis will be performed on survey items at the time of screening, including demographic survey, social history survey, vital signs, physical examination, medical history, surgical history evaluation, Brunnstrom stage (26), and K-MMSE (28) evaluation. In addition, for continuous variables, the number of participants, average, standard deviation, median, minimum, and maximum values, and for categorical data, frequency and fraction will be presented in both total and groups. Statistical significance of differences between groups will be analyzed using an independent two-sample *t*-test for continuous data (Wilcoxon rank sum test if the normality distribution assumption is not satisfied) and chi-square test for categorical data.

#### 2.8.3. Analysis of primary outcome

Descriptive statistics (number of participants, mean, standard deviation, median, minimum, and maximum) of the BBT values at T0 and T2 and the degree of BBT change between T0 and T2 will be presented by group. The statistical significance of BBT changes between groups will be confirmed by analysis of covariance (ANCOVA) with the number of BBTs at T0 and stratified variables (depending on whether the UE-FMA score is less than 28 points) and occupational therapy dosage between T1 and T2 as covariates. Additionally, in the subgroup analysis, the location of stroke lesions will also be included as a covariate in the ANCOVA. All statistical analysis will be performed with significance level of 0.05.

#### 2.8.4. Analysis of secondary outcome

Descriptive statistics (number of participants, mean, standard deviation, median, minimum, and maximum) for BBT values at T0 and T1 and the degree of BBT changes between T0 and T1 will be presented by group. The statistical significance of BBT changes between groups will be confirmed by analysis of covariance (ANCOVA) analysis with number of BBTs at T0 and stratified variables (depending on whether the UE-FMA score is less than 28 points) as covariates.

For other secondary outcomes (UE-FMA score, K-MBI, NIHSS, FTT score, Brunnstrom Stage, Grip strength), descriptive statistics (number of participants, mean, standard deviation, median, minimum, and maximum) at T0, T1, and T2 will be presented for each group. The statistical significance of BBT changes between T0 and T1 and T0 to T2 between groups will be confirmed by ANCOVA with the number of BBTs at T0 and stratified variables (depending on whether the UE-FMA score was less than 28 points) and occupational therapy dosage during the corresponding period as covariates. Additionally, in the subgroup analysis, the location of stroke lesions will be included as a covariate in the ANCOVA. All statistical analysis will be performed with significance level of 0.05.

##### 2.8.4.1. Definition of evaluation analysis group

The FA set is defined as all subjects who are randomly assigned to this clinical trial, subjected to a test device or false procedure at least once, and evaluated for effectiveness. The per-protocol (PP) set is defined as all subjects who had completed a clinical trial without protocol violation among the subjects included in the FA set. However, if dropouts or significant clinical trial plan violations do not affect the validity evaluation, they may be included as PP sets, which are discussed, determined, and documented prior to de-blinding for statistical analysis. An effectiveness analysis will be performed for each of the FA and PP sets; if the subjects, including the analysis group, are different, all results will be presented. The final validity evaluation will be based on FA results.

### 2.9. Participant safety and withdrawal

A few severe adverse effects can occur during rTMS. Common side effects are mild, such as headaches and rarely reported occurrences of accidental seizures or induced hypomania (8). When a participant complains of these symptoms, the researcher will provide the necessary treatment with intensive observation. The summary and analysis of Treatment Emergent Adverse Event (TEAE) that occurs during and after the application of rTMS, descriptive statistics (number of participants, incidence, and number of occurrences) for TEAE, Adverse Device Effect (ADE), and serious adverse event (SAE) during and after the application of rTMS for clinical trials by group will be presented, and the difference in ratio between groups will be analyzed using the Chi-square test or Fisher’s exact test. The number of patients, incidence, and occurrences of coded abnormalities are presented by group using MedDRA to code abnormalities (TEEAE), medical device abnormalities (ADE), and critical abnormalities (SAE) after the application of clinical trial medical devices. The TEAE, ADE, and SAE that occurred after rTMS application were coded according to SOC and PT using MedDRA, and the number of participants, incidence, and occurrence of coded abnormal cases will be presented by group. In addition, a detailed list of individual participants who experienced significant abnormalities will be presented.

Participants are allowed to leave the trial at any time. Participants could withdraw from the trial after informing the research team; they will be encouraged to visit the prescheduled follow-ups, even if they withdraw from the study for data collection.

## 3. Discussion

This study was designed as a multi-center, double-blind, randomized sham-controlled trial that aims to confirm the efficacy of low-frequency contralesional rTMS in the recovery of upper limb motor function in patients with subcortical or brainstem ischemic stroke. To ensure the rigor of the investigation, the participants will be stratified into two subgroups based on their initial severity. Moreover, the amount of OT and PT will be the same in the intervention and control groups during the intervention period, and the amount of total therapy will be recorded throughout the trial. By controlling and recording the potential cofounders of the motor recovery after stroke, the study results are anticipated to report high-quality evidence on the effectiveness of rTMS on motor recovery after stroke.

In this study, among various rTMS protocols, low frequency rTMS over the contralesional M1 will be applied in attempts to normalize imbalance of IHI by suppressing the over-inhibition of contralesional M1 toward ipsilesional M1 (9). In previous studies, low-frequency rTMS was often chosen over high-frequency rTMS because of the low risk of seizures in the healthy corticospinal system and its minimal effect on motor behavior (8, 10). However, previous results regarding the efficacy of low-frequency rTMS over the contralesional M1 for motor recovery in patients with acute and subacute stroke remain inconsistent. Khedr et al. (24) reported that low-frequency rTMS is more advantageous in recovering upper extremity function after stroke compared to high-frequency rTMS. Dafotakis et al. (29) also reported the possible beneficial effects of low-frequency rTMS on hand function recovery in patients with acute and subacute subcortical stroke. In contrast, other studies have reported no significant beneficial effects of low-frequency rTMS compared with sham rTMS (30, 31). In a meta-analysis, Hsu et al. (11) confirmed the effect of low-frequency rTMS on the recovery of upper extremity function in patients with stroke and reiterated the notion that post-stroke hemisphere competition is altered and that this imbalance can be resolved by reducing the cortical excitability of unaffected hemispheres through low-frequency rTMS.

Recently, it has been reported that the location of stroke lesions is a key factor in the efficacy of rTMS in upper limb motor recovery. Kim et al. (13) confirmed that the recovery of the upper extremities was significant only in patients without cortical invasion. Ameli et al. demonstrated that high-frequency rTMS on the ipsilesional M1 was effective only in patients with subcortical stroke and not in patients with additional cortical stroke (32). Nowak et al. (33) conducted cross-over investigation in patients with subacute subcortical stroke and found that application of rTMS to the contralesional M1 improved the kinematics of finger and grasp movements in the affected hand. Based on these findings, the target population of this trial was narrowed down to patients with subacute subcortical or brainstem ischemic stroke. Previous studies have explained possible reasons for the better effects of rTMS in patients with stroke without cortical involvement. First, the expected increase in cortical activity in the ipsilesional M1 through rTMS is inefficient in propagating to other nodes of the motor network for motor recovery, as there is a structural or functional disconnection in stroke that invades the cortex (32, 34). Second, in the cortical brainstem, GABAergic intracortical inhibition is greater, and concurrent downregulation of GABA receptors in both hemispheres has been identified, which may be associated with the reduced effects of rTMS (13).

We also stratified the participants into two subgroups based on the initial UE-FMA score because it has been reported that rTMS can be less effective in patients with stroke with severe upper extremity paralysis. Ludemann-Podubeck et al. (35) insisted that when a larger part of the M1 or its corticospinal projections is involved in stroke, causing a more profound clinical hand motor deficit, the contralesional dorsal premotor cortex may have a positive influence on motor recovery. Therefore, low-frequency rTMS of the contralesional M1 may not help to restore functionality (35). Two other studies referred to the same mechanism and explained the correlation between the degree of paralysis and the effect of rTMS (36, 37). Based on these results, we excluded patients with stroke with extremely severe upper extremities paralysis (UE-FMA score of 15 or less and those with a wrist score of less than 1 and a hand score of less than 1) and stratified into two subgroups (UE-FMA score of 28 or higher and UE-FMA score below 28) to ensure that the initial severity in both the intervention group and control group is evenly distributed.

In this clinical trial, the change in the number of BBT at 4 weeks after EOT compared with that at baseline has been set as the primary efficacy evaluation variable. The BBT has been used in various rehabilitation studies since 1994, when its reliability and validity were verified as measures of hand function in the elderly (38); it is often used as a clinical variable to confirm hand function status and total agility of the upper limb, especially in patients with stroke (3). Accordingly, BBT has also been used as an effectiveness evaluation variable in various studies to evaluate the recovery of upper limb motor function using rTMS in patients with stroke (3, 5, 13, 18). The UE-FMA is more useful in assessing abnormalities of movement, whereas the BBT is more suitable for evaluating activity limitations (39). However, with UE-FMA, it is difficult to confirm the response to the treatment of patients with stroke with mild upper limb hemiplegia due to the ceiling effect. Therefore, in this study, the primary outcome measurement has been chosen as the change in the BBT.

This study has a few limitations. First, in the control group, a sham coil will not be used for sham rTMS. Instead, the real coil will be positioned perpendicular to the scalp with an intensity of 100% of the patient’s RMT, and the frequency and position will be the same as those in the intervention group. Although the electrical field induced by this sham stimulation method is lower and less focal than that of real stimulation (40), we cannot rule out the possibility that this low electric field strength may influence the modulation of cortical neurons. However, it has been reported that the intensity of sham stimulation is significantly weaker than that of real stimulation, suggesting a low likelihood of producing similar physiological effects as actual stimulation (41). To ensure the blindedness of the participants during intervention, the position of the coil will be placed and removed by the researcher to ensure that the participants cannot see the position of the coil throughout the intervention. Moreover, as all the participants in this study are patients with first-time stroke, there is little possibility that they could guess the group assigned to them because they may not have encountered rTMS equipment before. Second, in this study, a navigation system will not be used to determine the motor hotspots, which requires a separate MRI sequence for treatment and is difficult to apply in real clinical settings. We will use the Motor Evoked Potentials (MEP) of the First Dorsal interossei (FDI) muscle to determine motor hotspots because it has been reported that the method of finding the rTMS motor hotspot by MEP of the FDI muscle may be more effective than navigated rTMS (42).

## 4. Conclusion

This study is expected to provide evidence of the efficacy of upper limb motor function recovery using contralesional low-frequency rTMS in patients with subcortical and brainstem ischemic stroke.

## Ethics statement

The studies involving humans were approved by institutional review boards of Seoul National University Bundang Hospital and Chungnam National University Hospital. The studies were conducted in accordance with the local legislation and institutional requirements. Written informed consent for participation in this study was provided by the participants’ legal guardians/next of kin.

## Author contributions

MKS, SJ, WKC, W-SK, and N-JP conceived and coordinated the study. H-MC, SC, MKS, SJ, WKC, W-SK, and N-JP designed the study. WKC and N-JP administered the project. H-MC drafted the manuscript. All authors participated in reviewing and editing the manuscript. All authors contributed to the article and approved the submitted version.

## Funding

This work is supported by the Research Fund of Remed (Seongnam-si, Republic of Korea). Remed has no role in the design and conduct of the study; collection, management, analysis and interpretation of the data. The research team has full autonomy in all aspects of the study.

## Conflict of interest

The authors declare that the research was conducted in the absence of any commercial or financial relationships that could be construed as a potential conflict of interest.

## Publisher’s note

All claims expressed in this article are solely those of the authors and do not necessarily represent those of their affiliated organizations, or those of the publisher, the editors and the reviewers. Any product that may be evaluated in this article, or claim that may be made by its manufacturer, is not guaranteed or endorsed by the publisher.
